# CME ACTIVITY: Transmission of Human Papillomavirus in Heterosexual Couples

**DOI:** 10.3201/eid1406.070616.1

**Published:** 2008-06

Medscape, LLC is pleased to provide online continuing medical education (CME) for this journal article, allowing clinicians the opportunity to earn CME credit. Medscape, LLC is accredited by the Accreditation Council for Continuing Medical Education (ACCME) to provide CME for physicians. Medscape, LLC designates this educational activity for a maximum of 1.0 *AMA PRA Category 1 Credits*™. Physicians should only claim credit commensurate with the extent of their participation in the activity. All other clinicians completing this activity will be issued a certificate of participation. To participate in this journal CME activity: (1) review the learning objectives and author disclosures; (2) study the education content; (3) take the post-test and/or complete the evaluation at **http://www.medscape.com/cme/eid**; (4) view/print certificate.

## Learning Objectives

Upon completion of this activity, participants will be able to:

- Identify the most common baseline human papillomavirus (HPV) status of couples
- Specify the most common mode of transmission of HPV between couples
- Describe the role of anatomic sites in the transmission of HPV
- Identify behavioral factors associated with the transmission of HPV

## Editor

**D. Peter Drotman, MD**, Editor-in-Chief, *Emerging Infectious Diseases*. *Disclosure: D. Peter Drotman, MD, has disclosed no relevant financial relationships.*

## CME AUTHOR

**Charles P. Vega, MD**, Associate Professor; Residency Director, Department of Family Medicine, University of California, Irvine, California, USA. *Disclosure: Charles P. Vega, MD, has disclosed that he has served as an advisor or consultant to Novartis, Inc.*

## AUTHORS

Disclosures: **Brenda Y. Hernandez, PhD, MPH**; **Lynne R. Wilkens, DrPH**; **Xuemei Zhu, MD**; **Pamela Thompson, MPH**; **Katharine McDuffie, BS**; **Yurii B. Shvetsov, PhD**; **Jeffrey Killeen, MD**; **Lily Ning, MD**; and **Marc T. Goodman, PhD, MPH**, have disclosed no relevant financial relationships. **Lori E. Kamemoto, MD, MPH**, has disclosed that she has received grants for clinical research from GlaxoSmithKline, and is on the speakers’ bureau for Merck.

## Earning CME Credit

To obtain credit, you should first read the journal article. After reading the article, you should be able to answer the following, related, multiple-choice questions. To complete the questions and earn continuing medical education (CME) credit, please go to **http://www.medscape.com/cme/eid**. Credit cannot be obtained for tests completed on paper, although you may use the worksheet below to keep a record of your answers. You must be a registered user on Medscape.com. If you are not registered on Medscape.com, please click on the New Users: Free Registration link on the left hand side of the website to register. Only one answer is correct for each question. Once you successfully answer all post-test questions you will be able to view and/or print your certificate. For questions regarding the content of this activity, contact the accredited provider, CME@medscape.net. For technical assistance, contact CME@webmd.net. American Medical Association’s Physician’s Recognition Award (AMA PRA) credits are accepted in the US as evidence of participation in CME activities. For further information on this award, please refer to http://www.ama-assn.org/ama/pub/category/2922.html. The AMA has determined that physicians not licensed in the US who participate in this CME activity are eligible for *AMA PRA Category 1 Credits*™. Through agreements that the AMA has made with agencies in some countries, AMA PRA credit is acceptable as evidence of participation in CME activities. If you are not licensed in the US and want to obtain an AMA PRA CME credit, please complete the questions online, print the certificate and present it to your national medical association.

## CME Questions

1. Which of the following was the most common human papillomavirus (HPV) status at baseline in the current study cohort?
  A. Both partners HPV-negative
  B. One partner HPV-negative and one HPV-positive
  C. Both partners HPV-positive with the same HPV type
  D. Both partners HPV-positive with different HPV types
2. What was the most common means of HPV transmission in the current study?
  A. Male-to-female transmission
  B. Female-to-male transmission
  C. Male auto-inoculation
  D. Female auto-inoculation
3. Which of the following statements about anatomic sites of transmission of HPV in the current study is *most* accurate?
  A. Most women obtained infection from the glans of the penis
  B. The female anus was not a significant site of transmission to men
  C. There were no cases of transmission from the women’s hands to the men’s genitals
  D. Among men, the rate of auto-inoculation was comparable to the rate of transmission from women
4. Which of the following factors from the current study was *most* significant in the risk for HPV transmission?
  A. Frequency of condom use
  B. Length of relationship
  C. Any history of anal intercourse
  D. A history of genital herpes

**Table:** Activity Evaluation

**1. The activity supported the learning objectives.**				
Strongly Disagree				Strongly Agree
1	2	3	4	5
**2. The material was organized clearly for learning to occur.**				
Strongly Disagree				Strongly Agree
1	2	3	4	5
**3. The content learned from this activity will impact my practice.**				
Strongly Disagree				Strongly Agree
1	2	3	4	5
**4. The activity was presented objectively and free of commercial bias.**				
Strongly Disagree				Strongly Agree
1	2	3	4	5

